# Association between Obesity and Serum 25(OH)D Concentrations in Older Mexican Adults

**DOI:** 10.3390/nu9020097

**Published:** 2017-01-31

**Authors:** Victoria G. Rontoyanni, Jaqueline C. Avila, Sapna Kaul, Rebeca Wong, Sreenivas P. Veeranki

**Affiliations:** 1 Metabolism Unit, Shriners Hospitals for Children, 815 Market Street, Galveston, TX 77550, USA; virodoyi@utmb.edu; 2 Department of Surgery, University of Texas Medical Branch, 301 University Blvd, Galveston, TX 77555, USA; 3 Department of Preventive Medicine and Community Health, University of Texas Medical Branch, 301 University Blvd, Galveston, TX 77555, USA; jaqcontr@utmb.edu (J.C.A.) sakaul@utmb.edu (S.K.); rewong@utmb.edu (R.W.)

**Keywords:** Body Mass Index (BMI), serum 25(OH)D, vitamin D, obesity, older Mexican adult, Mexican Health and Aging Study (MHAS)

## Abstract

Background: Vitamin D is essential for maintaining bone mineralization and calcium homeostasis, and prevents falls and fractures in older adults. Mexico is undergoing an epidemiologic and demographic transition with increasing obesity rates. The study’s aim was to determine the association of obesity with serum 25-hydroxyvitamin D [25(OH)D] concentrations in older Mexican adults. Methods: Data from 1772 Mexicans, aged ≥50 years, enrolled in a sub-sample of the 3rd wave of the Mexican Health and Aging Study, were included. Serum 25(OH)D concentrations were used to define vitamin D status, and were categorized into tertiles. Body mass index measures were used to categorize older adults into under/normal weight, overweight, and obese groups. Multinomial logistic regression models were used to assess the relationship, adjusting for potential confounders. Results: Approximately 40% and 37% of older Mexican adults were either overweight or obese, respectively. Compared to under/normal weight older Mexicans, obese adults were 1.78 times (95% Confidence Interval (CI) 1.27–2.48) and 1.94 times (95% CI 1.40–2.68) more associated with the first and second tertile concentrations of serum 25(OH)D, respectively. Overweight adults were 1.52 times (95% CI 1.12–2.06) more associated with the second tertile of serum 25(OH)D concentration than under/normal weight adults. Conclusion: Overweight/Obesity was found to be significantly associated with low concentrations of serum 25(OH) in older Mexican adults.

## 1. Introduction

Vitamin D has garnered significant attention in the scientific and public health community, with an estimated one billion individuals of all ages and ethnicities currently being either insufficient or deficient worldwide [1,2,3], with the prevalence possibly being underestimated in older adults, who may also have lower serum 25-hydroxyvitamin D [25(OH)D] concentration [4]. Vitamin D is a fat-soluble vitamin, essential for maintaining extracellular calcium ion and phosphate levels that are vital for bone mineral homeostasis [5,6]. In addition to its role in skeletal health, epidemiological studies suggest that vitamin D plays a vital role in non-skeletal health outcomes [2,7,8], where low serum concentrations were found to be associated with cancer [9], autoimmune diseases [10,11], cardiovascular disease [12], diabetes [13,14,15], and other conditions such as muscle weakness [16], schizophrenia [17], depression [18,19], cognitive deficits [20,21], and fractures and falls [22,23,24,25]. Although the evidence for its role in non-skeletal health is inconsistent, inconclusive for causality, and insufficient to inform nutritional requirements [26], it appears prudent to maintain sufficient vitamin D levels in humans throughout their life course. 

Vitamin D is produced in the skin from the precursor 7-dehydrocholesterol, upon exposure to solar ultraviolet B radiation at wavelengths between 290 and 315 nm, but is also obtained from the diet and from dietary supplements. Via two hydroxylations, vitamin D derived from the skin or diet converts to 25-hydroxyvitamin D [25(OH)D] in the liver, and to its biologically active form, 1,25-dihydroxyvitamin D, in the kidney [2,27]. Several risk factors for vitamin D deficiency/insufficiency in older adults have been identified, including geographical latitude-dependent impaired skin synthesis, the season and time of the day, sunscreen use, skin pigmentation, dietary inadequacy or malabsorption, and liver or kidney dysfunction [2,27]. Another possible risk factor is obesity, as evidenced by epidemiological studies identifying an association between obesity and low vitamin D concentrations [28,29,30,31,32,33,34,35,36,37,38,39], possibly via the proposed action of excess body fat to sequester higher concentrations of serum 25(OH)D [31,40]. Additionally, impaired bioavailability of vitamin D, in response to ultraviolet radiation exposure, was observed in obese individuals, which is independent of the cutaneous precursor of vitamin D [31], possibly leading to low serum vitamin D concentrations.

In a meta-analysis of 21 observational studies of different age groups and populations, Pereira-Santos et al. (2015) demonstrated a 35% higher rate of vitamin D deficiency in obese individuals, than in those of a normal-weight [29]. However, 7 of these 21 observational studies found no association between vitamin D deficiency and obesity. Additionally, a majority of these studies were conducted in developed nations using Caucasian populations [29,30,31,32,33], with few studies conducted using Hispanic Americans [30,34,35,36], and very few studies using non-US Hispanics or Latino populations [37,38,39]. Middle-income countries like Mexico are undergoing an epidemiologic transition from infectious to non-communicable diseases with high rates of obesity [41]; in Mexico, more than a third of the population is currently obese [42,43,44]. Additionally, the rapid aging of the Mexican population increases the number of older adults who might possibly be at higher risk of vitamin D deficiency, and its skeletal and non-skeletal effects [4,45,46]. Thus, using a subsample (availability of biomarkers) of the 3rd Wave of the Mexican Health and Aging Study (MHAS), we hypothesized that vitamin D concentrations would be lower in overweight or obese older Mexican adults, when compared with their normal-weight counterparts. The aim of our study was to investigate the association between vitamin D status (quantified using serum 25(OH)D concentrations) and obesity (quantified using body mass index (BMI)) in older Mexican adults. 

## 2. Materials and Methods

### 2.1. 3rd Wave of the Mexican Health and Aging Study

The study population included participants enrolled in the 2012 cohort (Wave 3) of the MHAS, a nationally representative longitudinal cohort of older adults in Mexico. Details of the MHAS’s objective, aims, and design, have been published in earlier studies [47,48,49,50]. In brief, the cohort consists of individuals aged ≥50 years from both rural and urban areas of all 32 federal entities/states, and their spouses/partners of any age, if residing in the same household. The baseline interview was conducted in 2001, with follow-up interviews conducted in 2003 and 2012. During the 2012 follow-up survey, a total of 18,465 individuals were interviewed, including 12,569 follow-ups from 2001 and 2003 waves, and 5896 new respondents. Interviews were conducted during the months of October-December, and no specific information on study participants’ dietary intakes (foods and supplements), and sunlight exposure (effect of season, skin pigmentation, latitude), use of sunscreen lotions or creams, and skin pigmentation disorders, was obtained. Objective measures of anthropometrics, biomarkers, and performance tests, were collected from a sub-sample of 2086 direct respondents residing in four states, who were representative of the older adults in Mexico. In this sub-sample, serum 25(OH)D concentrations were measured for the first time in MHAS; thus was our study population. 

Of the 2,086 participants in the sub-sample, 156 individuals were found to <50 years old and non-representative of older adults in Mexico, and thus were excluded. Additional 158 older adults were excluded from the study due to missing information on study variables. There were no significant differences between participants with missing variables and those with non-missing data (data not shown), resulting in a final analytical sample of 1772 participants. The MHAS cohort was approved by the Institutional Review Boards of the University of Texas’ Medical Branch and the National Institute of Public Health in Mexico. The data used to conduct this study is de-identified, publicly available, and can be downloaded from the MHAS website [51].

### 2.2. Serum 25(OH)D Concentration Measures

An individual’s vitamin D status was assessed in serum by quantifying 25(OH)D concentration, which is an accurate indication of long-term vitamin D stores with a half-life of 2–3 weeks [52]. Venous blood samples of ~3 mL were collected in evacuated tubes, stored at 2 °C–8 °C, and centrifuged at 2500 rpm for 15–20 min, to produce nonhemolysed serum. The serum samples were deposited in the nitrogen tank and transported to the National Institute of Public Health in Cuernavaca for specific biological measurements. Serum 25(OH)D concentrations (total vitamin D2 and D3 concentrations) were measured on an automated analyzer (ARCHITECT System, Abbott Laboratories, Chicago, IL, USA), using a one-step delayed chemiluminescent microparticle immunoassay with an automated pretreatment step (3L52 ARCHITECT 25-OH vitamin D assay, Abbott Laboratories). As per the manufacturer’s values, the coefficient of variation (within-run and total) for 25(OH)D was ≤10% [53], and the limit of detection was ≤10.0 ng/mL [53]. Details about the biological sample collection, transportation and storage, and vitamin D assay test, were described in the MHAS Manual of Procedures [53]. Since there are no specific cut off values for serum 25(OH)D concentrations in obese individuals [54], we categorized serum 25(OH)D concentrations into tertiles. The first tertile included serum 25(OH)D concentrations ≤20.4 ng/mL, the second tertile included values between 20.5 and 26.6 ng/mL, and the third tertile included concentrations of 26.7ng/ml and above. Drawing comparisons with vitamin D cut-off points, defined by Institute of Medicine (IOM) and the Endocrine Society, the MHAS participants in the first tertile 25(OH)D group can be defined as being deficient, as per Endocrine Society Clinical Practice Guidelines (<20 ng/mL) [55], and both deficient (<12 ng/mL) and inadequate (12–20 ng/mL), as per IOM-defined categories [54]. 

### 2.3. Obesity-Body Mass Index Measures

BMI was used to define obesity. BMI was calculated as weight (in kg) divided by height (in m) squared. Weight and height were measured using an electronic portable scale to the nearest 0.1 kg and a stadiometer to the nearest 0.1 cm, respectively [53]. Consistent with the cutoff point values recommended by the Centers for Disease Control and Prevention [56], we categorized BMI into the following three categories: under/normal weight (<25.0 kg/m^2^); overweight (25–29.9 kg/m^2^); and obese (≥30.0 kg/m^2^). There were 14 cohort participants who were underweight (BMI <18.5 kg/m^2^), so we combined them into the “normal-weight” category. The waist circumference (WC), which is an indicator of abdominal obesity, was measured to the nearest 0.1 cm, using a fiberglass tape measure and standard techniques, as described in the MHAS Manual of Procedures [53]. Based on the World Health Organization recommendations for WC cut-off points indicating overweight and obesity [57], and similar to an earlier study in older Mexican adults [58], we categorized the study participants as overweight/obese if their WC was >102 cm in men and >88 cm in women. Since BMI and WC are highly correlated (R^2^ = 0.82, *p* < 0.001), and the majority of prior studies examined the association between BMI and circulating 25(OH)D concentration, we used BMI as the primary study exposure for making comparisons with earlier studies. 

### 2.4. Covariates 

Based on the available literature [30,37,59,60,61,62,63], several factors that might possibly influence the relationship between obesity and serum 25(OH)D concentrations were included. Information on demographic characteristics included sex (male/female), age at survey administration (50–59, 60–69, 70+ years), region of residence (semi-urban: population between 0–99,999, urban: population >100,000), marital status (single, married/living with a partner, divorced/separated, widowed), and health insurance status [Mexican Social Security Institute (IMSS), Institute for Social Security and Service for State Workers (ISSSTE), Seguro Popular, Other, Uninsured]. Physical activity (exercise or hard physical work ≥3 times/week), smoking (no/yes), and diagnosis of diabetes using hemoglobin A1C (HbA1C) levels (normal ≤5.7%, pre-diabetic = 5.7%–6.4%, and diabetic ≥6.4%), were included as additional covariates. 

### 2.5. Ethical Approval

This article includes data analysis of the Mexican Health and Aging Study (MHAS). The MHAS study involves human subjects, and the protocol is in accordance with the ethical standards of the Institutional Review Boards of the University of Texas Medical Branch in Galveston, TX and of the National Institute of Public Health in Mexico, and with the 1964 Helsinki declaration and its later amendments, for comparable ethical standards. 

### 2.6. Statistical Analysis

Descriptive statistics were used to present the characteristics of the cohort participants. Categorical variables were presented using frequencies and proportions, and compared using a χ^2^ test. Continuous variables were presented using mean values (±SD), and compared using *t* or one-way ANOVA tests. The study outcome was serum 25(OH)D concentrations categorized into three tertiles. The third tertile (highest serum 25(OH)D concentration) was used as a reference category in the regression model. Study exposure was obesity categorized into three groups using BMI levels. A multinomial logit model, which simultaneously estimates binary logits for all possible comparisons among outcomes with more than two categories, was used to assess the association of obesity with serum 25(OH)D concentrations, adjusting for confounders including sex, age, marital status, health insurance status, region of residence, smoking, physical activity, and diagnosis of diabetes mellitus. Additionally, to test for the interaction effect of sex on the relationship, we included an interaction term of sex with BMI categories in the models. Regression diagnostics were performed for each model and no collinearity was identified that warranted omission of variables. All analyses were conducted using Stata S.E. 14.0 (StataCorp, College Station, TX, USA). Two-sided statistical significance was considered for alpha at the 0.05 level.

## 3. Results

Table 1 presents the descriptive characteristics of the 1772 older adults included in the study cohort, and the statistical differences in sociodemographic, lifestyle, and health conditions between the three serum 25(OH)D concentration tertile groups. The average age of participants at the time of the survey was 63.4 years (±9.47 years). Overall, 58.6% of the older adults were female, 24.6% were >70 years old, 32.4% were either single, divorced/separated, or widowed, 15% were uninsured, 38.7% were smokers, 44.6% were physically active for ≥3 times/week, and 37.3% were diabetic. When studying participant characteristics between the three categories of serum 25(OH)D concentrations, significant differences were identified among several characteristics, except for smoking status (*p* = 0.5). Approximately 69% and 62% of older adults in the first and second serum 25(OH)D tertiles were female, respectively, compared to 46% in the third tertile (*p* < 0.001). Approximately 35% of participants in the third tertile had Seguro Popular insurance, compared to 24% and 27% of participants in the first and second tertiles (*p* = 0.001), respectively. More than half of the participants in the third tertile were physically active, compared to 35.4% and 46.7% of participants in the first and second tertiles (*p* < 0.001), respectively. Approximately 46% and 36% of participants in the first and second tertiles were diabetic, respectively, compared to 30% in the third tertile (*p* < 0.001). 

Overall, 39.9% and 36.8% of older adults were overweight and obese, respectively. Sixty-two percent of study participants were at a high risk of abdominal obesity (waist circumference measures). Obesity (defined using BMI) differed significantly between the three tertiles of serum 25(OH)D concentrations. Approximately 80% of older adults in both the first and second tertiles of serum 25(OH)D were overweight/obese, compared with 70% of participants in the third tertile (*p* < 0.001). 

Table 2 presents the results of the relative risk ratios (RRRs) of the association between obesity and serum 25(OH)D concentrations in older Mexican adults. Compared to older Mexican adults with under/normal weight, those who were obese were significantly associated with a 1.78 times (95% Confidence Interval [CI] 1.27–2.48) and a 1.94 times (95% CI 1.40–2.68) increased risk of serum 25(OH)D concentrations in the first and second tertiles, respectively. Additionally, older Mexican adults who were overweight were 1.52 times (95% CI 1.12–2.06) more likely to be in the second tertile than under/normal weight adults. There was no significant interaction between BMI levels and sex on serum 25(OH)D concentration (data not shown). Waist circumference and BMI were highly correlated (R^2^ = 0.82, *p* < 0.001). Sensitivity analyses of the association of obesity with 25(OH)D concentrations revealed similar estimates when obesity was defined using WC cut-off levels (data not shown).

## 4. Discussion

Using a sub-sample of the MHAS cohort, we found that overweight or obese older Mexican adults were associated with lower concentrations of serum 25(OH)D, when compared to their normal-weight counterparts. To our knowledge, this is the first study to investigate the association between obesity and vitamin D status in older Mexican adults. Given the epidemiological disease transformation (communicable to non-communicable) and demographic transformation (young to aging adults) in Mexico, with high rates of obesity, the study findings provide evidence for public health professionals and policy makers to address the obesity and vitamin D deficiency/insufficiency epidemics in Mexico, by regular screening of vitamin D concentrations in older adults, specifically targeting those who are obese or overweight. 

Approximately 33% of our study participants were identified as having low serum 25(OH)D concentrations (first tertile: ≤20.4 ng/mL or 51 nmol/L), in agreement with a previous study that reported vitamin D deficiency rates [serum 25(OH)D) cut-off: <50 nmol/L] among older Mexican adults of ~37% [64], signifying the need for nutrient supplementation to prevent immediate risk of falls and fractures, and late consequences of chronic comorbid conditions [65,66,67]. Based on the IOM definitions of vitamin D deficiency and insufficiency, 4% of older Mexican adults in our study were found to be vitamin D “deficient” (<12ng/mL or <30 nmol/L), and 32% as “insufficient” (<20 ng/mL or <50 nmol/L) [54]. More than two-thirds of older adults in our study were vitamin D insufficient, if the cut-off value of <30 ng/mL (<75 nmol/L) is used, as recommended by the American Geriatrics Society, the Endocrine Society, and the International Osteoporosis Foundation (IOF) [55,68,69,70]. Due to such inconsistencies, we categorized serum 25(OH)D concentrations into tertiles. These health agencies recommend a minimum serum 25(OH)D concentration of 30 ng/mL (75 nmol/L) for older adults, in order to prevent falls, and an estimated average vitamin D requirement of 20–25 µg/day (800–1000 IU/day), to reach the minimum concentration. Additional intake as high as 50 µg/day (2000 IU/day) is recommended for older adults who are either obese, have osteoporosis, limited sun exposure, or have malabsorption [68,69,70]. Thus, it is important that public health professionals, geriatricians, and policy makers identify the need for routine surveillance for serum 25(OH)D concentrations and adhere to IOF recommendations of 800–1000 IU of vitamin D supplementation to older Mexican adults who have insufficient vitamin D levels. 

Approximately 77% of older adults in our study were either overweight or obese, which is similar to the estimates for Mexican adults reported by the Food and Agriculture Organization Of The United Nations (FAO), the Organisation for Economic Co-operation and Development (OECD), and the National Health and Nutrition Interview Survey of Mexico [42,43,44,71]. Our estimate is in agreement with a prior estimate of the World Health Organization’s (WHO) Study on global AGeing and adult health (SAGE), which used a representative sample of 2032 older Mexicans and found that 78% were classified as overweight or obese [72]. If no nation-wide interventions and policies are put in place, it is projected that the current rates of overweight/obesity in adult males and females will rise to 88% and 91%, respectively, by 2050 [73]. Therefore, Mexican public community and policy makers should develop and implement effective public health interventions and policies in order to halt the increasing trend in obesity prevalence in Mexico. 

The epidemiological relationship between obesity and vitamin D is complex, and potentially bidirectional. A study using data on genetic markers explored the causality and direction of the relationship between BMI and 25(OH)D, and found that a higher BMI leads to lower 25(OH)D concentrations [28]. In contrast, the contribution of 25(OH)D to increased BMI was found to be small. Similarly, clinical trials that used weight-loss interventions reported an increase in serum 25(OH)D concentrations post-intervention [74,75,76]. Vitamin D is fat-soluble and thus, is readily stored in adipose tissue [77,78], with excess body fat being expected to store higher concentrations of serum 25(OH)D. Prior studies have demonstrated enhanced vitamin D uptake by the adipose tissue [31,40], and >50% decreased bioavailability of cutaneous vitamin D in obese subjects [31]. This might possibly explain the low concentrations of serum 25(OH)D in the obese individuals in our study. In contrast, there is evidence for high 25(OH)D concentrations stimulating lipogenesis and inhibiting lipolysis, resulting in increased triglyceride stores in obese adults [79,80,81]. Since our study is cross-sectional in nature, and limits causality and directionality, longitudinal aging cohort studies should further assess the relationship between obesity and vitamin D in older adults. 

This study is subject to limitations that merit discussion. We used cross-sectional data from a subsample of the 2012 Wave of MHAS, which limits the causal relationship of obesity with 25(OH)D concentrations in older Mexican adults. Information on MHAS study participants’ dietary intakes (foods and supplements), sunlight exposure (effect of season, skin pigmentation, latitude), use of sunscreen lotions or creams, and skin pigmentation disorders, was not available, and future studies should adjust for the confounding role of these variables. Similarly, no previous history of hepatic or renal disorders, anticonvulsant medications, or corticosteroids use was available, which might influence vitamin D bioavailability. Although we adjusted for several confounders, there is a possibility for residual confounding. Furthermore, our study was limited in addressing the clinical significance of the identified association between lower serum 25(OH)D concentrations and obesity. Our study’s findings do not absolutely reflect the idea that increasing vitamin D concentrations will either treat or prevent obesity, and future clinical trials are needed to address the beneficial effects of vitamin D intake on clinical outcomes in older Mexican adults.

## 5. Conclusions

In summary, the current study identified that obese or overweight older Mexican adults had an increased risk of low concentrations of serum 25(OH)D, when compared to their under/normal weight counterparts. Given the epidemiological transition in Mexico, with its rapid aging [45], rising prevalence of obesity, and vitamin D inadequacy/insufficiency among older Mexican adults, concerns of a heightened risk of osteoporosis and associated fractures [2,25,82], and associated healthcare costs in Mexico over the next few years, are considerable [83]. Mexican public health experts and policy makers should develop interventions for addressing the vitamin D deficiency among older adults, specifically among obese or overweight adults, and reduce the healthcare burden in Mexico. 

## Figures and Tables

**Table 1:** Characteristics of study participants by serum 25(OH)D concentration tertiles in a sub-sample of 3rd Wave of Mexican Health and Aging Study, *n* = 1772.

Characteristic	Study Cohort	Serum 25(OH)D			*p*-Value
		First Tertile (≤ 20.4 ng/mL)	Second Tertile (20.5–26.6 ng/mL)	Third Tertile (≥26.7 ng/mL)	
	*n* (%)	%	%	%	
Sex					
Male	733 (41.4)	31.5	38.3	54.3	<0.001
Female	1039 (58.6)	68.5	61.7	45.7	
Age (in years)					
50–59	738(41.6)	33.7	43.4	47.9	<0.001
60–69	599 (33.8)	35.2	32.7	33.5	
>70	435 (24.6)	31.0	23.9	18.7	
Marital status					
Single	91 (5.1)	7.1	4.4	3.9	0.001
Married	1198 (67.6)	61.6	69.9	71.5	
Divorced or Separated	171 (9.6)	9.7	8.5	10.7	
Widowed	312 (17.6)	21.6	17.2	13.9	
Insurance					
Uninsured	266 (15.0)	15.3	14.8	14.9	0.001
IMSS ^a^	682 (38.5)	40.9	38.7	35.8	
ISSSTE ^b^	180 (10.2)	12.9	10.6	7.0	
Seguro Popular	508 (28.7)	23.9	27.1	35.0	
Other	136 (7.8)	6.9	8.9	7.3	
Region of residence					
Semi-urban	750 (42.3)	30.4	44.6	52.2	<0.001
Urban	1022 (57.7)	69.6	55.4	47.8	
Smoking					
Yes	686 (38.7)	37.3	38.3	40.6	0.5
No	1086 (61.3)	62.8	61.7	59.4	
Physical activity ^c^					
Yes	790 (44.6)	35.4	46.7	51.8	<0.001
No	982 (55.4)	64.6	53.3	48.2	
Hemoglobin A1c					
Normal	399 (22.5)	18.5	22.8	26.3	<0.001
Pre-Diabetes	713 (40.2)	35.7	41.4	43.6	
Diabetes	660 (37.3)	45.8	35.8	30.1	
Body Mass Index					
Under/Normal weight	412 (23.3)	20.8	19.6	29.4	<0.001
Overweight	708 (39.9)	38.1	40.5	41.3	
Obesity	652 (36.8)	41.1	39.9	29.4	
Waist circumference					
Low risk	673 (38.0)	30.0	33.9	50.1	<0.001
High risk	1099 (62.0)	70.0	66.1	49.9	

Bolding indicates statistical significance on the chi-square test at *p*-value < 0.05. ^a^ IMSS: Mexican Social Security Institute. ^b^ ISSSTE: Institute for Social Security and Service for State Workers. ^c^ Exercise or hard physical work ≥3 times/week

**Table 2:** Relationship between obesity and serum vitamin D concentrations in older Mexican adults in a subsample of 3rd wave of Mexican Health and Aging Study, *n* = 1772.

Body Mass Index	Unadjusted		Adjusted ^b^	
	First Tertile RRR ^a^ (95% CI) ^c^	Second Tertile RRR ^a^ (95% CI) ^c^	First Tertile RRR ^a^ (95% CI) ^c^	Second Tertile RRR ^a^ (95% CI) ^c^
Under/Normal weight	Ref	Ref	Ref	Ref
Overweight	1.30 (0.97–1.75)	1.47 (1.10–1.98)	1.34 (0.97–1.84)	1.52 (1.12–2.06)
Obesity	1.98 (1.46–2.67)	2.03 (1.50–2.77)	1.78 (1.27–2.48)	1.94 (1.40–2.68)

Bolding indicates statistical significance at *p*-value < 0.05. ^a^ RRR stands for relative risk ratio. ^b^ Models adjusted by sex, age, marital status, health insurance status, region of residence, smoking, physical activity, and HbA1c. ^c^ 95% confidence interval
